# Positive Youth Development and Mental Well-Being in Late Adolescence: The Role of Body Appreciation. Findings From a Prospective Study in Norway

**DOI:** 10.3389/fpsyg.2021.696198

**Published:** 2021-08-23

**Authors:** Helga Bjørnøy Urke, Ingrid Holsen, Torill Larsen

**Affiliations:** Department of Health Promotion and Development, University of Bergen, Bergen, Norway

**Keywords:** body appreciation, mental well-being, positive youth development, adolescence, body image, PYD five Cs

## Abstract

Although a vulnerable period, adolescence is a time of great potential for healthy development. Understanding factors that contribute to mental well-being in this period is of great importance to facilitate for healthy development. During the adolescence period the body goes through rapid and considerable changes, and the focus on body appearance and perfection is substantial at societal, media, and peer level. In this study, we investigated the association between dimensions characterizing positive youth development, and mental well-being among Norwegian adolescents 16–17 years 1 year later, and whether this association was mediated by degree of body appreciation. We further tested whether the indirect and direct paths between positive youth development dimensions and mental well-being were moderated by gender. We used data from the first and second follow-up of the control arm of the COMPLETE study, a cluster randomized controlled trial in upper secondary schools in Norway. Findings showed that positive youth development in grade 1, as measured by both a combined global PYD and the dimensions competence, and connection were significantly associated with mental well-being in grade 2 in models adjusted for mental well-being in grade 1, gender and perceived family affluence. Also, body appreciation in grade 1 significantly predicted mental well-being in grade 2 in models including each of global PYD, competence, connection, character, and caring dimensions. Contrary to our hypothesis, no mediating effects of body appreciation were observed, and no moderation of indirect effects by gender were observed.

## Introduction

Adolescents experience a range of social, psychological, and physical transitions that prepare and shape them for young adulthood. For example, the body goes through rapid and considerable changes, and the focus on body appearance and perfection is substantial at societal, media, and peer level. Although a vulnerable period, adolescence is a time of great potential for positive and healthy development (Gilman and Huebner, 2003). During the past decades we have seen a growing interest in positive youth development (PYD) as an asset- and resource based approach toward understanding youths’ development through adolescence (Lerner et al., 2005, 2009). The theoretical and empirical field of positive youth development (PYD) has contributed substantially to balancing the focus and knowledge on the adolescent years as not only a period of distress and risk, but also as a period of great potential for positive development, thriving and well-being. A related, but largely separate field of research is that of positive body image, which also has been associated with positive notions of body and well-being (Swami et al., 2018; Davis et al., 2020). In the present study, we are particularly concerned with individual psychosocial resources for mental well-being in the period of late adolescence (16- to 17-year-olds), of which positive body image may be one. We conceptualize mental well-being broadly, including both hedonic and eudemonic aspects reflecting subjective experiences of life satisfaction and happiness, and psychological functioning – in line with other scholars in the field of positive mental health (Ryan and Deci, 2001; Tennant et al., 2007). In the present study, we highlight recent developed and validated positive constructs reflecting positive processes and factors in PYD, body appreciation and mental well-being. We aim to contribute to a broader and deeper understanding of the mechanisms that contribute to forming mental well-being in the late adolescence period through a focus on the role of PYD dimensions as well as body appreciation as one aspect of positive body image.

### PYD as a Resource for Mental Well-Being

The five Cs of PYD – *Competence, Confidence, Connection, Character*, and *Caring –* is a theoretically and empirically supported framework in the field of PYD research (Lerner et al., 2005). The five Cs are associated with positive development outcomes both when considered as separate constructs and combined in a higher-ordered Global PYD measure (Geldhof et al., 2015; Holsen et al., 2017; Gomez-Baya et al., 2019; Tomé et al., 2021). *Competence* reflects academic, social, and vocational skills (Lerner et al., 2005, 2009). It is a central factor in self-regulation and a basic psychological need as postulated in Self-determination theory (Deci and Ryan, 2000). *Confidence* reflects having a sense of mastery, positive identity and positive self-worth (Lerner et al., 2005, 2009) and a general positive self-esteem (Birkeland et al., 2014). Further, positive self-esteem is a strong predictor of psychological well-being (Diener, 2009). *Connection* reflects having healthy relations with community, friends, family and school (Lerner et al., 2005, 2009), and feeling connected is strongly linked to subjective well-being. Research has found that acceptance from peers had a generally protective effect on global self-esteem among youth (Birkeland et al., 2014) and is crucial for young people’s positive development (Settertobulte and Matos, 2004). Opposite, those who are not socially connected to friends are more likely to have problems related to mental health (Settertobulte and Matos, 2004). *Character* reflects having integrity, moral commitment, personal values, interpersonal values and skills, and respect for societal and cultural rules (Lerner et al., 2005, 2009). An important part of character building is the feeling of having influence over decisions and co-determination in one’s own life. *Caring* reflects having empathy and sympathy for others (Lerner et al., 2005). Caring refers to both caring for others and being cared for by others and is closely linked to the feeling of belonging and connected (Lerner et al., 2005). It is important for the individual’s mental health to be seen and to experience caring from others (Anvik and Gustavsen, 2012). The Global PYD measure has in recent research been found to be associated with positive outcomes, such as life satisfaction (Shek and Chai, 2020), internal self-regulation (Gestsdottir et al., 2017), and contribution (Geldhof et al., 2014). Although previous research indicates that both the global PYD measure and the separate dimensions predict later advantageous outcomes, few studies have looked at the association with subsequent subjective mental well-being as conceptualized in this study. Based on previous research, we hypothesize that PYD is associated with later mental well-being among Norwegian adolescents. Specifically:

*1. Levels of PYD indicators, both global PYD and separate dimensions, in first grade of upper secondary school are positively associated with levels of mental well-being in second grade of upper secondary school after adjusting for first grade levels of mental well-being, gender, and socioeconomic position.*

### The Role of Body Appreciation for Mental Well-Being

Similar to the change from a dominating risk perspective of the adolescence period to the field of PYD, research on *body image* has also transitioned from an almost exclusive pathogenic approach focusing on negative dimensions (i.e., body dissatisfaction) and negative health outcomes, to also including a salutogenic or resource based focus on body image, such as body appreciation or overall positive body image (Avalos et al., 2005; Tylka and Wood-Barcalow, 2015a). How youth perceive their body and physical appearance has been demonstrated to be of major importance to psycho-social indicators such as self-esteem, optimism (e.g., Avalos et al., 2005) and social relationships (e.g., Holsen et al., 2012), which are important aspects also addressed within PYD theory. Though research on positive body image is much less developed compared to research on negative body image, this subfield has grown in the past decade contributing to a less fragmented understanding of body image among youth. The increasing amount of research has manifested that positive body image is not merely the opposite of negative body image or body dissatisfaction, but that a positive body image is a unique concept, and important to study and promote in itself (Avalos et al., 2005; Tylka and Wood-Barcalow, 2015a,b). For example, in body image therapy you cannot focus only on reducing body dissatisfaction. Just as important is a focus on enhancing body appreciation (Tylka and Wood-Barcalow, 2015a). Tylka (2011) claims that there are reasons to believe that factors affecting us positively such as positive connections with other people may have a greater impact on well-being than factors affecting us negatively, indicating that facilitating adolescents’ resources for body appreciation and later mental well-being is equally or even more important than trying to prevent risk. Positive body image is multifaceted in nature, and include broad conceptualizations of body, body appreciation, and body acceptance, to name a few (Tylka and Wood-Barcalow, 2015a). As such, including the field and concepts of positive body image in the field of PYD broadly, and mental well-being specifically, can enrich our understandings of how the established PYD dimensions, e.g., the five Cs, are connected to positive aspects of body image and well-being.

A few studies have investigated the association between a positive body image and various indicators of wellbeing. Swami et al. (2018) found positive associations between several aspects of a positive body image and emotional, psychological, and social wellbeing in British adults. Other studies have found associations between body appreciation and self-compassion (Homan and Tylka, 2015), life satisfaction (Davis et al., 2020), lower levels of depression and higher levels of self-esteem (Gillen, 2015; Baceviciene and Jankauskiene, 2020), and views on body functionality (Baceviciene and Jankauskiene, 2020). Most of these studies were, however, conducted with United States college samples (Gillen, 2015), or other adult samples (Homan and Tylka, 2015; Davis et al., 2020). Since 2017, we identified four studies which have thoroughly validated the body appreciation scale among adolescents in countries in Europe and Asia (Lemoine et al., 2018; Baceviciene and Jankauskiene, 2020; Razmus et al., 2020; Maes et al., 2021). One of these have examined the association between body appreciation and well-being (Lemoine et al., 2018), among adolescents, 12 to 19 years of age, in Denmark, Portugal, and Sweden. Similar results were found in the three countries, with body appreciation being positively correlated with self-esteem and psychological well-being.

As seen from the recent published studies just referred to, research on positive body image and mental well-being among youth outside of the United States context is increasing but is still scarce. It is not necessarily so that the life situation for young people in various western countries are the same. For example, Norway has a relatively homogeneous population of young people, with immigrant children accounting for 6.8% and children with immigrant parents accounting for 11.8% of the 0-to-17 years age group (Statistics Norway, 2019). Norway also has well-developed welfare services and benefits with a strong public sector and broad social security system for all. Education is free from grade one through college/university and is a right for all youth. The majority of adolescents attend public upper secondary schools, and schools are therefore central institutions both for the individual students and their community for stimulating to positive development.

According to several national surveys, most Norwegian children and youth report being generally satisfied and content with their life (FHI, 2018). However, life satisfaction decreases as children grow older (from 11 to 16 years of age) (FHI, 2018). This decrease could potentially be explained by the parallel increase in psychological distress, which is more frequent among girls than boys (FHI, 2018). Less is known about levels and the influencing role of positive body image among adolescents in Norway, but one recent study focusing on positive aspects of body among college students found overall relatively high scores on body appreciation (Sundgot-Borgen et al., 2021). Considering both potentially context specific aspects that could influence the relationships in question, and the importance of more knowledge on this topic in general, the Norwegian context is relevant to study. Additionally, most research on the association between body appreciation and mental well-being indicators have hitherto been based on cross-sectional data, and studies with a prospective design are needed to understand the role of body appreciation for mental well-being in the adolescent period.

In this study, we aim to contribute with empirical knowledge on the aspect of *body appreciation* and *mental well-being*, focusing on Norwegian youth in upper secondary school. Based on the limited existing research, we hypothesize that

*2. Levels of body appreciation in first grade of upper secondary school are positively associated with levels of mental well-being in second grade of upper secondary school after adjusting for first grade levels of mental well-being, gender, and socioeconomic position.*

### Body Appreciation as Mediator Between PYD and Mental Well-Being

Tylka and Wood-Barcalow (2015b) discuss positive body image in relation to a broad conceptualization of beauty. For example, people may include inner characteristics when describing beauty, such as confidence and caring for others (Tylka and Wood-Barcalow, 2015a). Hence, inhabiting perceptions and values of confidence and caring may contribute to a higher value of own body, which in turn may contribute to higher general well-being. Likewise, the development of character includes the development of critical reflection which might be of importance for developing a healthy body image in the face of external influences like peer pressure, and stereotypical body images frequently seen in social- and other media, which may in turn have an impact on well-being. In a qualitative study (Frisén and Holmqvist, 2010), Swedish adolescents expressed that beauty was not about fitting with a societal ideal, but more about ‘being yourself.’ In the five Cs PYD framework, as outlined above, PYD is theorized as comprised of competence, confidence, connection to others, character, and a caring dimension (Lerner et al., 2005). Following from the theoretical assumptions by Tylka and Wood-Barcalow (2015a) and other scholars, having high scores on these constructs may lead to a greater appreciation of own body, which in turn contributes to better general well-being. To explore the role of body appreciation in the relationship between PYD constructs and mental well-being, we included body appreciation as a mediator between PYD in grade 1 (both global PYD and the separate indicators) and mental well-being in grade 2. Even if body appreciation may partially mediate the effect of PYD indicators on subsequent mental well-being, other variables may also transmit the effect of PYD onto mental well-being. Thus, in line with recommendations for partial mediation (Holland et al., 2017), we include a direct effect to capture this unmodeled effect, as well as account for mental well-being levels in grade 1 and socioeconomic position. We hypothesize that:

*3. The association between positive youth development in first grade of upper secondary school and mental wellbeing in the second grade of upper secondary school is partially mediated by body appreciation in first grade of upper secondary school, when controlling for mental well-being at T1, gender, and socioeconomic position.*

### Gender as a Moderator of the Relationships Between PYD, Body Appreciation, and Mental Well-Being

**FIGURE 1:**
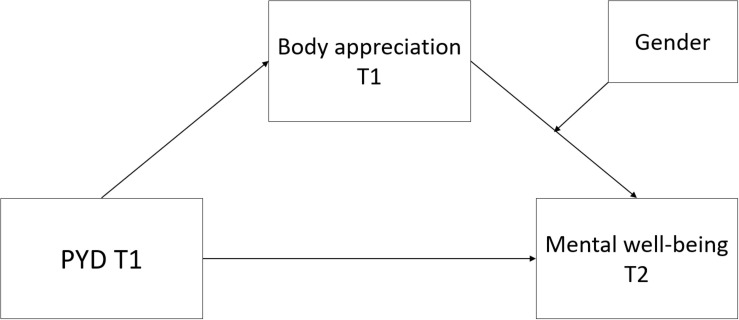
The conceptual second stage moderated mediation model.

The role of gender in these relationships is relevant to consider (Gillen, 2015). Several studies have investigated gender differences in body appreciation. In their meta-analysis, He et al. (2020) conclude that males generally report higher levels of body appreciation compared to women, but they also highlight age as one moderating variable to this difference as women’s body appreciation seem to increase with age, but is relatively stable among men. In line with these findings, a recent study of Norwegian college students found slightly higher mean scores on body appreciation for men compared to women (Sundgot-Borgen et al., 2021). Considering the potential role of age, it could be that greater differences exist in lower age groups. Lemoine et al. (2018) observed that adolescent boys reported slightly, but statistically significantly higher levels of body appreciation compared to adolescent girls across three countries. When it comes to the association between body image and mental well-being, existing research largely indicates that there are not significant gender differences. Gillen (2015) found in her study of undergraduate US college students that associations between a positive body image and indicators of health were not dependent on gender. This is similar to other recent studies (Lemoine et al., 2018; Davis et al., 2020; Sundgot-Borgen et al., 2021) finding that body appreciation predicted or correlated with life satisfaction or psychological well-being in both adult women and men, and adolescent girls and boys, and any gender differences being marginal (Lemoine et al., 2018). However, since most of the empirical literature on gender differences in body appreciation and mental-well-being is with (young) adults, the present study adds to this literature with the perspective from late adolescence in a prospective study examining more complex models. We do not hypothesize a difference in a specific direction but take an exploratory approach to gender (Figure 1).

*4. We explore gender as moderating the path between body appreciation in the first grade of upper secondary school, and mental well-being in the second grade of upper secondary school, controlling for mental well-being in first grade, and socioeconomic position.*

## Materials and Methods

### Data

This study used data from the first (T1) and second (T2) follow-up of the control arm of the COMPLETE study, a cluster randomized controlled trial in upper secondary schools in Norway (Larsen et al., 2018). The sample included adolescents from five public upper secondary schools spread across four counties in Norway, covering urban, semi-urban, and rural areas. In our sample, the age range was 16–25, and respondents above 19 years at T1 (*n* = 41) were excluded from the data material to comply with the WHO definition of adolescents as people between 10 and 19 years of age (WHO, n.d.). The final study sample was *N* = 659 at T1, mean age *M* = 16.9 (SD = 0.44) with a gender distribution of 45.1% females and 54.9% males. At T1, 64.9% of the sample classified their family as economically *well-off*, 27.6% as *somewhat well off*, and 7.5% as *poorly off*. The total sample with data on both T1 and T2 was 348. Mean age at T2 was *M* = 17.84 (SD = 0.38) with a gender distribution of 42% females and 58% males.

### Measures

**Mental well-being** was measured with the short version of the Warwick Edinburgh Mental Well-Being Scale (SWEMWBS) developed for use in population level surveys (Tennant et al., 2007). This measure is based on a broad and solely positive conceptualization of well-being that includes affective-emotional and cognitive-evaluative aspects, and psychological functioning (Tennant et al., 2007). The short version of the measure consists of seven items and participants are asked to respond what best describes their experience over the last 2 weeks on a five-point Likert scale ranging from 1 = *None of the time* to 5 = *All of the time*. The items are as follows: *I’ve been feeling optimistic about the future, I’ve been feeling useful, I’ve been feeling relaxed, I’ve been dealing with problems well, I’ve been thinking clearly, I’ve been feeling close to other people, and I’ve been able to make up my own mind about things.* The scale is previously validated among adolescents (McKay and Andretta, 2017), including in Norway (Ringdal et al., 2018). We used the translated version of Smith et al. (2017) (see details in their publication). Items were combined into a composite variable that was used in further analyses.

**Body appreciation** was measured with the Body Appreciation Scale-2 (BAS-2) (Tylka and Wood-Barcalow, 2015b). This scale consists of 10 items and participants are asked to respond on a five-point Likert scale to what extent the 10 statements are true for them with responses 1 = *Never*, 2 = *Seldom*, 3 = *Sometimes*, 4 = *Often*, and 5 = *Always*. Sample items include *I feel good about my body*, and *I appreciate the different and unique characteristics of my body*. Since its development, the BAS-2 has been validated in many contexts (Tylka and Wood-Barcalow, 2015b; Alleva et al., 2016; Lemoine et al., 2018; Junqueira et al., 2019), but to the best of our knowledge, not in Norway. We used the method of forward and back translation to translate the BAS-2 measure. First, two researchers fluent in Norwegian and English independently translated the original items from English to Norwegian. Next, another two researchers also fluent in Norwegian and English independently translated the items back to English. Finally, through a discussion among the four researchers it was concluded on a synthesized final version of the items to be included in the questionnaire.

**Positive Youth Development** was measured with the short form of the PYD five Cs model (PYD-SF). The original measure includes 34 items across the five dimensions (Cs) *connection, confidence, competence, caring*, and *character* (Geldhof et al., 2014) partly based on several already established scales (Harter, 1988; Eisenberg et al., 1996; Benson et al., 1998; Davis et al., 2020; Wichstrøm, 1995). Items were rescaled to 0–12 in line with the recommended scoring protocol (Lerner, 2010). The PYD-SF measures had previously been used in a Norwegian context and were based on the translation done in this previous work (Holsen et al., 2017).

*Competence* was measured with six items covering academic, social, and physical self-perceived competence based on the existing Norwegian version of Harter’ Self-Perception Profile for Adolescents (Harter, 1988) developed by Wichstrøm (1995). Participants responded on a four-point Likert scale, ranging from 1 = *Really true for me* to 4 = *Really not true for me.* Examples of items are *I feel I am just as smart as others at my age* (academic), *I have a lot of friends* (social), *I think I can do well at just about any new athletic activity* (physical).

*Confidence* was measured with six items covering self-worth, physical appearance, and positive identity, again based on Wichstrøm’s (1995) Norwegian version of Harter’s Self-Perception Profile for Adolescents (Harter, 1988). Participants responded on the same four-point Likert scale as for Competence. Sample items include *I think I am good looking*, and *All in all, I am glad I am me*.

*Connection* was measured with eight items covering the youth’s relationship to family, peers, school, and neighborhood. Participants responded on a five-point Likert scale, ranging from 1 = *Strongly agree* to 5 = *Strongly disagree* for the domains of family, school, and neighborhood, and from 1 = *Almost never true* to 5 = *Always true* for the peer domain. Sample items include *I get a lot of encouragement at my school* (school), *My friends care about me* (peer).

*Character* was measured with eight items covering personal values and social consciousness from Search Institute Profile of Student Life: Attitudes and Behaviors (Benson et al., 1998). Participants responded on a five-point Likert scale ranging from 1 = *Not important* to 5 = *Extremely important.* Sample items include *Doing what I believe is right even if my friends make fun of me* (personal values) and *Giving time and money to make life better for other people* (social consciousness).

*Caring* was measured with six items covering empathy and sympathy based on the Eisenberg Sympathy Scale (Eisenberg et al., 1996) and the empathic concern subscale of the Interpersonal Reactivity Index (Davis, 1983). Participants were asked to respond on a five-point Likert scale according to how well each statement fit with their perception of themselves. Response options ranged from 1 = *Not well* to 5 = *Very well.* Sample items include *When I see someone being taken advantage of, I want to help them*, and *It makes me sad to see a person who doesn’t have friends.*

Gender (male/female) was based on registry data retrieved from school registries (Larsen et al., 2018), and included as control and moderator variable (male = 0 and female = 1) in analyses.

A proxy for socioeconomic position was measured with a one item measure of perceived family affluence that is considered valid for measuring adolescents’ perception of own socioeconomic position relative to others and is for example used in the international WHO Health Behavior in School Children study to assess socioeconomic status (Pförtner et al., 2015). Participants were asked to answer the question: *How well off is your family?* Response options were on a five-point Likert scale ranging from 1 = *Not at all well off* to 5 = *Very well off*. This variable was modified to three categories combining the two lowest categories and the two highest categories, resulting in the categories 1 = *Not well off*, 2 = *Somewhat well off*, and 3 = *Well off*.

### Analyses

Statistical analyses were performed in IBM SPSS and R. Descriptive statistics were obtained for all study variables. To assess the construct validity of our measures CFA was conducted, relying on the following model fit estimates with recommended cut-offs: CFI > 0.95, RMSEA < 0.05, and SRMR < 0.05 indicating good model fit, and CFI > 0.90, RMSEA < 0.08, and SRMR < 0.08 indicating acceptable model fit (Hooper et al., 2008). Further measurement invariance tests across gender (and time points for mental well-being) were conducted for all main study variables to assess validity of the measures using the same fit criteria. Internal consistency was assessed for all measures, using the omega coefficient (ω). Upon creation of sum scores Independent sample’s *t*-tests were conducted to assess mean differences in the main study variables by gender. Further, Spearman’s correlation analyses were conducted between all study variables stratified by gender. Next, six sets of hierarchical regression models were conducted, assessing the role of PYD indicators (global and separate dimensions) and body appreciation at T1 as predictors of mental well-being at T2 adjusting for gender, perceived family affluence, and mental well-being at T1. For each set of models, at step 1 the PYD indicator was included, at step 2, body appreciation was introduced, and at step 3, the control variables gender, perceived family affluence, and mental well-being at T1 were introduced. To test our Hypothesis 3, a set of mediation models were carried out examining any indirect effect of PYD indicators at T1 on mental well-being at T2 through body appreciation at T1, with gender, perceived family affluence, and mental well-being at T1 as covariates of mental well-being T2. Lastly, a set of second stage moderated mediation models were conducted with gender as the moderation variable of the indirect relationships between PYD indicators at T1 and mental well-being at T2 through body appreciation at T1.

Missing data was handled with pairwise deletion (bivariate and regression analyses) and FIML (for mediation and moderated mediation analyses).

### Ethics

The COMPLETE study was approved by the Norwegian Centre for Research Data. For further details, see Larsen et al. (2018).

## Results

### Construct Validity and Measurement Invariance

Confirmatory factor analyses (CFA)s were conducted based on the translated sets of the original items for the SWEMWBS, PYD-SF, and BAS-2. For SWEMWBS the model did not achieve acceptable fit at T1 (CFI = 0.967, RMSEA = 0.109, SMRM = 0.025) or at T2 (CFI = 0.956, RMSEA = 0.131, SMRM = 0.033). Examining modification indices, it was decided to allow correlated error terms in a stepwise manner for the following two pairs of items *I’ve been feeling optimistic about the future*, and *I’ve been feeling useful*, and *I’ve been feeling close to other people* and *I’ve been able to make up my own mind about things* for T1, achieving acceptable fit (CFI = 0.995, RMSEA = 0.046, SRMR = 0.014). For T2, it was decided to allow correlated error terms in a stepwise manner for the following two items *I’ve been feeling optimistic about the future*, and *I’ve been feeling useful*, and *I’ve been feeling useful*, achieving acceptable fit (CFI = 0.991, RMSEA = 0.064, SRMR = 0.021). Other validation studies in the Norwegian context have made very similar adjustments to the SWEMBS (Smith et al., 2017; Ringdal et al., 2018). In the present study, the internal consistency of the measures was ω 0.95 and 0.95 at T1 and T2, respectively.

CFA for PYD was first conducted for each of the separate indicators. Except for caring (CFI = 0.992, RMSEA = 0.063, SRMR = 0.020), none of the separate indicators achieved acceptable fit (Competence: CFI = 0.701, RMSEA = 0.218, SRMR = 0.094; Confidence: CFI = 0.701, RMSEA = 0.218, SRMR = 0.094; Connection: CFI = 0.582., RMSEA = 0.280, SRMR = 0.147; Character: CFI = 0.750, RMSEA = 0.147, SRMR = 0.077). For competence, examination of modification indices resulted in correlating of error terms in a stepwise manner, three in total: *I have a lot of friends* and *I do very well at my classwork*; *I feel I am just as smart as others at my age* and *I think I can do well at just about any new athletic activity*; and *I do very well at my classwork* and *I am better than others my age at sports*, resulting in acceptable fit (CFI = 0.985, RMSEA = 0.059, SRMR = 0.034). For confidence, when examining the correlation matrix some very high correlations were observed (>0.7). When assessing the wordings of highly correlated items, some were very similar indicating redundancy. It was decided to remove challenging items. This was one item with high estimates for several error term correlations: *I am happy with myself most of the time*, and one item with low loadings: *I do things I know I should not do*. The latter is not surprising as it is a negatively worded item which often shows to influence model fit negatively. The fit for the adjusted measure was good (CFI = 0.999, RMSEA = 0.027, SRMR = 0.009). For connection, examination of modification indices resulted in stepwise correlation of error terms for the item pairs for connection to peers, family, and community. Specifically, correlation of error terms were done for items *I feel my friends are good friends* and *My friends care about me*, items *I have lots of good conversations with my parents* and *In my family I feel useful and important*, items *Adults in my town or city make me feel important* and *Adults in my town or city listen to what I have to say*, upon acceptable fit was achieved (CFI = 0.976, RMSEA = 0.075, SRMR = 0.075). For character, examination of modification indices resulted in stepwise correlation of error terms for the following two pairs of items *Knowing a lot about people of other races* and *Enjoying being with people who are of a different race than I am*, and for *Helping to make the world a better place to live in* and *Giving time and money to make life better for other people*, upon good fit was achieved (CFI = 0.978, RMSEA = 0.046, SRMR = 0.031).

In the present study, all the PYD constructs showed good internal consistency as measured with the omega (ω) coefficient, ranging from 0.81 to 0.95 (see Table 1). Items were then combined into composite indicators for each of the five Cs constructs as well as a general PYD indicator.

**TABLE 1:** Descriptive statistics for study variables: Means, standard deviations, skewness, kurtosis, omega coefficients, and intercorrelations for study variables for boys and girls.

Variable	*N*	*M*	*SD*	Min, Max	Skewness (SE)	Kurtosis (SE)	ω	1	2	3	4	5	6	7	8	9
1. Mental well-being T2	312	3.50	0.89	1, 5	−0.47 (0.14)	0.39 (0.28)	0.95	–	0.54**	0.35**	0.47**	0.38**	0.48**	0.45**	0.24**	0.22**
2. Mental well-being T1	487	3.49	0.89	1, 5	−0.35 (0.11)	−0.03 (0.22)	0.95	0.53**	–	0.54**	0.62**	0.46**	0.48**	0.58**	0.44**	0.28**
3. Body Appreciation Scale T1	486	3.64	1.02	1, 5	−0.52 (0.11)	−0.41 (0.22)	0.98	0.42**	0.53**	–	0.62**	0.48**	0.63**	0.44**	0.44**	0.34**
4. General PYD T1	460	8.27	1.55	3.38, 11.91	−0.23 (0.11)	−0.08 (0.23)	0.92	0.43**	0.65**	0.59**	–	0.66**	0.69**	0.75**	0.75**	0.68**
5. Competence T1	515	7.41	2.27	0, 12	−0.38 (0.11)	0.04 (0.22)	0.85	0.36**	0.53**	0.43**	0.68**	–	0.60**	0.43**	0.30**	0.21**
6. Confidence T1	501	8.00	2.64	0, 12	−0.51 (0.11)	−0.15 (0.22)	0.93	0.45**	0.63**	0.74**	0.75**	0.58**	–	0.52**	0.38**	0.22**
7. Connection T1	505	8.92	1.96	2.63, 12	−0.52 (0.11)	0.01 (0.22)	0.90	0.42**	0.54**	0.45**	0.76**	0.43**	0.53**	–	0.45**	0.36**
8. Character T1	503	8.09	1.86	1.5, 12	−0.53 (0.11)	0.39 (0.22)	0.81	0.14	0.25**	0.14*	0.59**	0.18**	0.18**	0.30**	–	0.58**
9. Caring T1	521	8.65	2.93	0, 12	−1.03 (0.11)	0.61 (0.21)	0.95	0.10	0.15**	0.13	0.51**	0.13	0.10	0.26**	0.61**	–

**p < 0.01, *p < 0.05.

*Correlation coefficients presented above and below the diagonal for boys and girls, respectively. Gender code: 0 = boys, 1 = girls.*

CFA on the original items was conducted for BAS-2, not achieving acceptable fit (CFI = 0.957, RMSEA = 0.121, SRMR = 0.027). Examining the modification indices, the item *I am attentive to my body’s needs* had very high estimates in several error term correlations, and comparatively slightly lower loadings than the other items. However, it was decided to keep the item for external validity purposes and pursue correlation of error terms. Examination of modification indices resulted in stepwise correlation of six error terms for the following pairs of items *I feel good about my body* and *I am attentive toward my body’s needs, I am comfortable in my body* and *I feel like I am beautiful even if I am different from media images of attractive people (e.g., models, actresses/actors), I take a positive attitude toward my body* and *My behavior reveals my positive attitude toward my body; for example, I hold my head high and smile, I feel love for my body* and *I appreciate the different and unique characteristics of my body, I appreciate the different and unique characteristics of my body* and *I feel like I am beautiful even if I am different from media images of attractive people (e.g., models, actresses/actors), I feel good about my body* and *My behavior reveals my positive attitude toward my body; for example, I hold my head high and smile.* Acceptable fit was achieved after this process (CFI = 0.982, RMSEA = 0.082, SRMR = 0.021). In the present study, the internal consistency of the modified version of BAS-2 was ω 0.98. Items were combined into a composite variable that was used in further analyses.

Next, we examined all constructs for measurement invariance across gender. Detailed results are presented in Supplementary Table 1. For SWEMWBS we reached partial strong invariance at T1 and T2. For BAS-2, we achieved strict invariance; for global PYD we achieved metric invariance, for competence, connection, and character we achieved partial strong invariance, and for confidence and caring, we achieved strong invariance. These analyses indicated that the measures included are invariant between boys and girls strengthening the confidence in the validity of the measures for use across gender.

### Descriptive Statistics

Table 1 presents descriptive statistics and omega estimates for each of the main constructs included in this study, as well as the bivariate correlations between each of the variables separately for boys and girls. As can be seen, the mean values for mental well-being and body appreciation approached 4 (on scales ranging 1–5), indicating relatively high mean body appreciation and high mean mental well-being. Similarly, mean values for the PYD dimensions were around 8 (on scales ranging 0–12), also indicating at least a skew toward high scores in these constructs.

Independent samples *t*-test showed statistically significant gender differences for all constructs, except for global PYD (*t* = 0.850) and connection (*t* = 0.235). Boys reported statistically significantly higher scores than girls on mental well-being (T1 *t* = 4.680; and T2 *t* = 3.294), body appreciation (*t* = 5.972), competence (*t* = 6.516), and confidence (*t* = 5.687), and girls reported statistically significantly higher scores than boys on character (*t* = −2.881) and caring (*t* = −6.779) (Table 2).

**TABLE 2:** Independent samples *t*-test statistics for study variables by gender.

	Boys			Girls				
		
Variable	*N*	*M*	*SD*	*N*	*M*	*SD*	*t*	Cohen’s *d*
1. Mental well-being T2	177	3.65	0.87	135	3.31	0.90	3.294***	0.38
2. Mental well-being T1	260	3.67	0.90	227	3.29	0.84	4.680***	0.43
3. Body Appreciation Scale T1	262	3.89	0.93	224	3.35	1.04	5.972***	0.54
4. General PYD T1	250	8.32	1.61	210	8.20	1.46	0.850	0.08
5. Competence T1	288	7.97	2.25	227	6.71	2.09	6.516***	0.58
6. Confidence T1	274	8.61	2.35	227	7.28	2.79	5.687***	0.52
7. Connection T1	276	8.94	1.98	229	8.89	1.94	0.235	0.03
8. Character T1	272	7.87	2.00	231	8.34	1.66	−2.881**	0.25
9. Caring T1	291	7.94	3.20	230	9.55	2.24	−6.779***	0.57

***p < 0.001, **p < 0.01.

*Gender code: 0 = male, 1 = female.*

Correlation analyses split by gender showed statistically significant associations between all main study variables with medium to strong effect sizes for most pairs (Cohen, 1988). Correlation analyses showed that girls and boys demonstrated somewhat differing relationships between the constructs. For example, while for boys the relationships between body appreciation and character and caring, respectively, were statistically significant with medium (*r* = 0.44) to small (*r* = 0.34) correlation coefficients, for girls these were significant at *p* < 0.05 with a small correlation coefficient (*r* = 0.14) and non-significant (*r* = 0.13), respectively. Similarly, the correlation coefficient for the relationship between body appreciation and mental well-being at T2 was *r* = 0.35 and *r* = 0.42 for boys and girls, respectively. These findings indicate that PYD may play a different role for boys and girls in the relationship between PYD indicators and body appreciation, and body appreciation and later mental well-being. However, when testing whether correlation coefficients were significantly different for boys and girls, these were found to be non-significant. We therefore moved on with regression analyses combined for girls and boys, controlling for gender in the regression models. However, since moderation effects can exist in more complex models, we pursued exploring the role of gender as a moderator in mediation models.

### Hierarchical Regression Analyses

Regression analyses served to test hypotheses 1 and 2. Results from the six sets of hierarchical regression models assessing the role of each PYD indicator separately (global and separate dimensions) and body appreciation at T1 for mental well-being at T2 are presented in Table 3. In all sets of models, the PYD indicator at T1 was positively and statistically significantly associated with mental well-being at T2 at step 1. When body appreciation was introduced in step 2, the coefficient of the PYD indicator was substantially reduced, but remained statistically significant for all models, except for models assessing the character (*p* = 0.194), and caring (*p* = 0.533) dimensions, indicating partial mediating effects of body appreciation. Body appreciation was also positively and statistically significantly associated with mental well-being at T2 in all unadjusted models (*p* values ranging from 0.000 to 0.005). At step 3, when control variables where introduced, most significantly mental well-being at T1, the PYD indicators were still statistically significant in the models assessing the role of global PYD [*b* = 0.10 (0.04), *p* = 0.018], competence [*b* = 0.06 (0.03), *p* = 0.032], and connection [*b* = 0.07 (0.03), *p* = 0.016], but not confidence [*b* = 0.04 (0.03), *p* = 0.100], character [*b* = 0.01 (0.03), *p* = 0.597], or caring [*b* = 0.01 (0.02), *p* = 0.575]. Our hypothesis 1, that PYD predicts subsequent mental well-being, was partly supported with results indicating that even when taking into account mental well-being levels at T1, adolescents’ global PYD levels, competence and connection with others had unique effects on later mental well-being. Similarly, and partially supporting our hypothesis 2, body appreciation remained statistically significant in all adjusted models [global PYD *b* = 0.13 (0.06), *p* = 0.034; competence *b* = 0.16 (0.06), *p* = 0.008; connection *b* = 0.12 (0.06), *p* = 0.047; character *b* = 0.16 (0.06), *p* = 0.008; and caring *b* = 0.16 (0.06), *p* = 0.009], except in that assessing the confidence dimension of PYD [*b* = 0.12 (0.07), *p* = 0.098], indicating that body appreciation plays a unique role in predicting mental well-being above and beyond mental well-being levels at T1 and important PYD dimensions of competence, connection, character and caring.

**TABLE 3:** Hierarchical regression model examining the prospective relationship between positive youth development, body appreciation, and mental well-being, adjusting for gender, perceived family affluence, and mental well-being at time 1.

Models and variables	*b* (SE)	*t*	*p*	*df*	Adjusted *R*^2^	*b* (SE)	*t*	*p*	*df*	Adjusted *R*^2^	*b* (SE)	*t*	*p*	*df*	Adjusted *R*^2^
**Model PYD total**															
PYD total T1	0.24 (0.03)	7.843	0.000	1, 275	0.180	0.15 (0.04)	4.190	0.000	2, 265	0.230	0.10 (0.04)	2.373	0.018		
Body Appreciation Scale T1						0.25 (0.06)	4.187	0.000			0.13 (0.06)	2.129	0.034		
Gender											−0.17 (0.09)	−1.847	0.066		
Perceived family affluence (ref poorly off)														6, 258	0.297
Somewhat well off											0.17 (0.19)	0.873	0.383		
Well off											0.13 (0.19)	0.716	0.475		
Mental well-being T1											0.31 (0.07)	4.499	0.000		
**Model PYD competence**															
PYD competence T1	0.15 (0.02)	6.658	0.000	1, 299	0.126	0.10 (0.03)	4.029	0.000	2, 281	0.201	0.06 (0.03)	2.156	0.032		
Body Appreciation Scale T1						0.26 (0.06)	4.693	0.000			0.16 (0.06)	2.662	0.008		
Gender											−0.10 (0.10)	−1.069	0.286		
Perceived family affluence (ref poorly off)														6, 272	0.244
Somewhat well off											0.20 (0.20)	1.000	0.318		
Well off											0.21 (0.20)	1.085	0.318		
Mental well-being T1											0.27 (0.07)	4.005	0.000		
**Model PYD confidence**															
PYD confidence T1	0.15 (0.02)	8.124	0.000	1, 295	0.180	0.09 (0.03)	3.624	0.001	2, 282	0.195	0.04 (0.03)	1.651	0.100		
Body Appreciation Scale T1						0.20 (0.07)	2.811	0.005			0.12 (0.07)	1.662	0.098		
Gender											−0.10 (0.09)	−1.091	0.276		
Perceived family affluence (ref poorly off)														6, 273	0.269
Somewhat well off											0.18 (0.19)	0.908	0.365		
Well off											0.19 (0.19)	1.032	0.303		
Mental well-being T1											0.34 (0.06)	5.312	0.000		
**Model PYD connection**															
PYD connection T1	0.18 (0.03)	7.014	0.000	1, 299	0.138	0.12 (0.03)	4.399	0.000	2, 287	0.190	0.07 (0.03)	2.421	0.016		
Body Appreciation Scale T1						0.25 (0.05)	4.666	0.000			0.12 (0.06)	1.991	0.047		
Gender											−0.20 (0.10)	−2.137	0.034		
Perceived family affluence (ref poorly off)														6, 279	0.253
Somewhat well off											0.19 (0.20)	0.977	0.330		
Well off											0.21 (0.19)	1.092	0.276		
Mental well-being T1											0.29 (0.07)	4.142	0.000		
**Model PYD character**															
PYD character T1	0.08 (0.03)	3.058	0.002	1, 298	0.027	0.034 (0.03)	1.303	0.194	2, 287	0.138	0.01 (0.03)	0.530	0.597		
Body Appreciation Scale T1						0.33 (0.05)	6.240	0.000			0.16 (0.06)	2.657	0.008		
Gender											−0.16 (0.10)	−1.673	0.096		
Perceived family affluence (ref poorly off)														6, 279	0.223
Somewhat well off											0.23 (0.20)	1.146	0.253		
Well off											0.27 (0.19)	1.409	0.160		
Mental well-being T1											0.33 (0.07)	5.025	0.000		
**Model PYD caring**															
PYD caring T1	0.04 (0.02)	2.169	0.031	1, 303	0.012	0.01 (0.02)	0.624	0.533	2, 288	0.138	0.01 (0.02)	0.561	0.575		
Body Appreciation Scale T1						0.34 (0.05)	6.702	0.000			0.16 (0.06)	2.647	0.009		
Gender											−0.18 (0.10)	−1.731	0.085		
Perceived family affluence (ref poorly off)														6, 279	0.223
Somewhat well off											0.24 (0.20)	1.193	0.234		
Well off											0.27 (0.19)	1.433	0.153		
Mental well-being T1											0.33 (0.07)	5.020	0.000		

Results from the mediation models are presented in Table 4 and show that none of the mediation paths were statistically significant when adjusting for mental well-being at T1, and thus, our hypothesis 3 was not supported.

**TABLE 4:** Direct and indirect effects from six separate models of PYD indicators at T1 (total and individual) on mental well-being at T2 including body appreciation at T1.

					Bootstrapping 95% CI	
					
	Effect	SE	*t*	*p*	Lower	Upper
**Total effect of PYD indicators T1 on mental well-being T2**						
PYD total	0.23	0.03	7.78	0.000	0.18	0.29
PYD competence	0.15	0.02	6.70	0.000	0.11	0.20
PYD confidence	0.14	0.02	8.05	0.000	0.11	0.18
PYD connection	0.18	0.03	7.25	0.000	0.13	0.23
PYD character	0.14	0.02	8.05	0.000	0.11	0.18
PYD caring	0.04	0.02	2.35	0.019	0.007	0.080

					**Bootstrapping 95% CI**	
					
	**Effect**	**SE**	***z***	***p***	**Lower**	**Upper**

**Direct effect of PYD indicators T1 on mental well-being T2**						
PYD total	0.10	0.04	2.48	0.013	0.017	0.180
PYD competence	0.06	0.03	1.79	0.073	−0.005	0.120
PYD confidence	0.05	0.03	1.56	0.120	−0.014	0.100
PYD connection	0.08	0.03	2.55	0.011	0.018	0.140
PYD character	0.05	0.07	1.55	0.120	−0.029	0.250
PYD caring	0.02	0.02	0.78	0.430	−0.023	0.054
**Indirect effect of PYD indicators T1 on mental well-being T2 through body appreciation at T1**						
PYD total	0.032	0.017	1.87	0.061	0.001	0.068
PYD competence	0.017	0.009	1.92	0.055	0.003	0.037
PYD confidence	0.024	0.017	1.40	0.160	−0.008	0.059
PYD connection	0.010	0.01	1.34	0.180	−0.001	0.029
PYD character	0.026	0.017	1.53	0.130	−0.007	0.059
PYD caring	0.007	0.005	1.47	0.14	0.000	0.018

*Note: All models adjusted for gender, perceived family affluence and mental well-being at T1.*

Given that previous research suggests that body appreciation might be influenced by gender and given that moderation of indirect effects can occur without a prior statistically significant indirect effect, it was decided to test a set of moderated mediation models with gender as moderator of the second stage of the mediation model, i.e., the path between body appreciation and mental well-being. No indication of moderated mediation with gender as the moderating variable was observed for the other PYD indicators (see details presented in Supplementary Tables 2–7).

## Discussion

In the present study, we took a comprehensive approach to the interlinkages between PYD, body appreciation, and mental well-being, and explored the role of gender in this system. Specifically, the objective was to assess the role of PYD (global and separate indicators), and body appreciation in the first grade of upper secondary school for mental well-being 1 year later in a sample of Norwegian adolescents. We hypothesized that PYD – combined and as separate dimensions of *competence, confidence, connection, character* and *caring –* and body appreciation predicted later better mental well-being (hypotheses 1 and 2), and that body appreciation mediated the relationship between PYD and mental well-being (hypothesis 3). We also explored the moderating role of gender in the mediation models.

In line with our hypothesis 1, findings revealed a statistically significant association between PYD as measured by global PYD, competence, and connection in the first grade of upper secondary school and mental well-being 1 year later. Feeling competent and connected to significant others are considered as two (of three) basic psychological needs (Ryan and Deci, 2001), which when fulfilled contribute to positive adjustment. As such, our findings are consistent with these theoretical assumptions. The lack of predictive influence of confidence, character, and caring indicates that the effect of global PYD was largely driven by the effect of competence and connection. The three other dimensions did not have unique significant contributions in adjusted models. This could be due to potentially less favorable consequences of having high scores on these constructs. For example, previous studies have shown that higher scores on caring (Holsen et al., 2017; Geldhof et al., 2019; Kozina et al., 2021), and character (Kozina et al., 2021) dimensions were associated with higher levels of symptoms of anxiety and depression. For the case of caring, this suggests that being too concerned, over-empathic or over-invested in others might result in negative effects on own mental health. For character, the concern with following rules and being evaluated by others have been discussed as elements that explain the relationship with anxiety (Kozina et al., 2021). As previously described by Geldhof et al. (2019) the mechanisms that underlie these observations need to be further examined. In the case of mental well-being, these previously described negative effects on psychological health could reduce the overall mental well-being of adolescents.

Consistent with our hypothesis 2, we identified statistically significant associations between body appreciation and mental well-being 1 year later in most of the fully adjusted models (global PYD, competence, character, and caring). This indicated that a stronger appreciative perception of own body may influence subsequent overall mental well-being in adolescence, even after controlling for levels of mental well-being at T1. This is in line with the body of existing literature on positive aspects of body and well-being (Swami et al., 2018; Davis et al., 2020). Other literature has also found associations between a positive body image and other perceptions of oneself, like self-compassion (Homan and Tylka, 2015) and self-esteem (Gillen, 2015; Lemoine et al., 2018). In our study, we saw significant correlations between body appreciation and all of the five PYD dimensions (competence, confidence, connection, character and caring), especially strong were the associations with competence and confidence, indicating that a generally positive self-perception may spill over to also appreciating one’s own body as suggested by Tylka and Wood-Barcalow (2015a). Further, the inclusion of body appreciation in the regression model resulted in a reduction in the coefficient size of PYD indicators, suggesting a potential mediating role of body appreciation in the relationship between PYD and mental well-being. In spite of the significant associations between PYD indicators, body appreciation and mental well-being, when testing our third hypothesis that body appreciation mediated the relationship between PYD (global and separate indicators) and mental well-being, this was not supported in any of our analyses. This could be due to these constructs measuring overlapping aspects that influence mental well-being more than having a mediating relationship. For example, the confidence measure includes aspects of physical appearance which could overlap with the body appreciation construct. Another plausible scenario is that the PYD indicators and body appreciation are interlinked, but not in a mediating manner, and their concurrent relationship was supported in our analyses.

We also explored the role of gender on the indirect and direct effects in a set of moderated mediation models. No specific hypothesis was posed for this due to limited empirical evidence, particularly the lack of evidence among men (He et al., 2020). Our findings did not support differing indirect effects depending on gender in the second stage moderated mediation models, for any of the PYD indicators (global or separate). Based on our preliminary bivariate analyses and significance tests of the difference in correlations between boys and girls for body appreciation and mental well-being, this was not surprising. Body appreciation was a consistent predictor of mental well-being across several models supporting that this relationship is strong for both boys and girls. This is also consistent with previous research which has indicated that the relationship between body appreciation and indicators of mental well-being are similar across boys and girls/men and women (Gillen, 2015; Lemoine et al., 2018). It was clear from regression analyses, that when including the control variable of mental well-being at T1, the coefficients of our PYD and body appreciation variables were substantially reduced, indicating that former mental well-being is a more important predictor of later mental well-being than former PYD or body appreciation.

Across our main study variables of mental well-being, body appreciation, and PYD indicators, mean levels were relatively high, indicating that youth in our sample are well adjusted, have appreciative body perceptions, and are generally satisfied with their situation. This is in line with other research, e.g., on adolescent life satisfaction across a range of contexts and countries (Gilman and Huebner, 2003). The mean scores of body appreciation in our study were very similar to those found in the recent study of Norwegian college students (Sundgot-Borgen et al., 2021), strengthening our confidence in knowledge on levels of this construct in the Norwegian setting. Compared to the recent study of adolescents in Denmark, Portugal, and Sweden (Lemoine et al., 2018), Norwegian adolescents reported consistently lower scores on body appreciation. This difference may be due to a narrower age range in our study (16–18 compared to 12–19 in Lemoine et al., 2018). The finding that levels of mental well-being in the first grade of upper secondary school predicted mental well-being in the second grade, underscores the value of creating environments that foster this positive outcome. Adding to that, the unique and significant role of both body appreciation and some of the PYD indicators in the final adjusted models point to these as indicators to focus on in creating such environments. Lastly, it seems like these relationships are similar for boys and girls.

### Limitations and Recommendations for Future Research

The present study has some limitations that must be noted. First, the sample size is relatively small, limiting the possibility of further relevant sub-group analyses (e.g., by socioeconomic status) and the inclusion of several control variables because of lack of statistical power. Third, the sample was drawn from schools that were self-selected into the COMPLETE study, and this limits generalizability to the general adolescent population. However, even if schools were self-selected, students were not until the next stage, which reduces the potential bias of self-selection happening at school level. Fourth, the sample of schools were limited to four out of 19 counties in Norway, and the sample is thus not nationally representative. The sample does, however, cover rural, semi-urban, and urban living areas in the north and west of the country, increasing representativeness of adolescent life in Norway. Fifth, due to challenges in achieving acceptable fit for some of the measures used in the study, it was decided to remove two items in the *confidence* dimension of PYD. This is not ideal as it could compromise our confidence in the validity of the measure. However, it was considered the most appropriate considering the values of these items in the CFA. The item *I am attentive to my body’s needs* in the BAS-2 measure was identified as potentially problematic for achieving acceptable fit in this sample. We decided to keep the item, but note that several previous studies have found this item to be problematic, e.g., with comparatively low loadings on the overall construct (Alleva et al., 2016; Kertechian and Swami, 2017; Swami et al., 2017; Lemoine et al., 2018), including among adolescents (Lemoine et al., 2018). Further examination of this item, e.g., in focus group discussions with adolescents is warranted.

Some of the mentioned limitations could be addressed in future research. For example, future studies exploring the role of body appreciation for mental well-being using longitudinal data with more time points could increase understanding of the potential causal and/or reciprocal nature of this relationship. Collecting data in large and representative samples would enable more meaningful subgroup analyses to be undertaken, additional confounding variables to be included, and increase robustness and generalizability of findings.

### Strengths and Implications

The study also holds several strengths worth emphasizing. First, the present study contributes to the limited knowledge of the role of an appreciative body image for mental well-being in the adolescent population. Very few studies have looked at these positive aspects generally, and even fewer in the adolescent population. Second, we examined these relationships using a prospective study design which gives a stronger basis for knowledge on causal links. Third, the study used validated measures to study these associations, which contributes to the validity and generalizability of the findings. We did thorough validation tests, e.g., testing for measurement invariance, with results supporting invariance across gender in the Norwegian context. Fourth, the focus on the mediating role of body appreciation in the relationship between PYD and mental well-being in late adolescence is innovative and adds to the field of PYD. This study provides some implications for practice. Considering that there is individual and societal value in facilitating and strengthening adolescents’ capacity for mental health and well-being, this study shows that one important arena for efforts is supporting adolescents’ body appreciation. This is particularly important considering the extreme focus on body appearance at societal level, and the vulnerable period that adolescence is for the development of body image (Littleton and Ollendick, 2003). Supporting adolescents’ appreciation of own body could be through redressing emphasis on body function, rather than physical – and body appearance in child and adolescent settings such as school, sports, and other leisure activity settings. Focus on body function has been found to spark more positive notions of own body, e.g., linked to gratitude, and to less self-objectification (Alleva et al., 2019). Further, research indicates that there is a reciprocity between individuals and their environment where individuals with a positive body image support and promote positive body image in others (Wood-Barcalow et al., 2010). Following, this type of positive embodiment ties closely to drawing on individual and contextual resources in line with PYD conceptualizations. The study findings support the value of a holistic approach to adolescent life that encompasses nurturing a range of domains of personal and social development.

In conclusion, our study explored new grounds for research on PYD by incorporating the field of positive body image into the PYD context. Although causal inferences cannot be made, the findings of this study indicate that (1) PYD, particularly *competence* and *connection*, seem to be important characteristics for subsequent mental well-being, and (2) an appreciative take on own body is associated with better mental well-being among adolescents 1 year later, which together suggests that facilitating for experiences that foster adolescents’ mastery and social networks, and supporting positive notions of adolescents’ body perceptions is important to facilitate overall and future mental well-being. Further, although no mediating role of body appreciation was observed, its influence on PYD indicator coefficients and associations with mental well-being show its importance as an asset or resource for mental well-being.

## Data Availability Statement

The raw data supporting the conclusions of this article will be made available by the authors, without undue reservation.

## Ethics Statement

The studies involving human participants were reviewed and approved by Norwegian Centre for Research Data. Written informed consent to participate in this study was provided by the participants’ legal guardian/next of kin when the participant was below 16 years.

## Author Contributions

HBU, IH, and TL conceptualized the study. HBU conducted analyses and drafted the first manuscript. All authors contributed to writing and interpreting results. All authors approved the final version of the manuscript.

## Conflict of Interest

The authors declare that the research was conducted in the absence of any commercial or financial relationships that could be construed as a potential conflict of interest.

## Publisher’s Note

All claims expressed in this article are solely those of the authors and do not necessarily represent those of their affiliated organizations, or those of the publisher, the editors and the reviewers. Any product that may be evaluated in this article, or claim that may be made by its manufacturer, is not guaranteed or endorsed by the publisher.
