# Supplementary material for: Positive Youth Development and Mental Well-Being in Late Adolescence: The Role of Body Appreciation. Findings From a Prospective Study in Norway
Source: Front Psychol. 2021 Aug 23;12:696198. doi: 10.3389/fpsyg.2021.696198 (PMC8419256; doi:10.3389/fpsyg.2021.696198)
Supplement: Supplementary file 1 [file Table_1.docx]

| **Supplementary Table 1. Measurement invariance (model fit statistics) of mental well-being at T1 and T2, body appreciation, and PYD indicators across gender** | | | | | | | | | |
| --- | --- | --- | --- | --- | --- | --- | --- | --- | --- |
| Model tested | *χ2* | *df* | RMSEA (90% CI) | CFI | SRMSR | ΔRMSEA | ΔCFI | ΔSRMSR | *p* |
| ***Mental well-being T1*** |  |  |  |  |  |  |  |  |  |
| Configural invariance | 29.532 | 24 | .037 (.000, .076) | .997 | .016 |  |  |  |  |
| Metric invariance | 33.479 | 30 | .025 (.000, .063) | .998 | .023 | .012 | .001 | .007 | .865 |
| Partial strong invariance | 36.902 | 34 | .021 (.000, .058) | .998 | .025 | .004 | .000 | .002 | .549 |
|  |  |  |  |  |  |  |  |  |  |
| ***Mental well-being T2*** |  |  |  |  |  |  |  |  |  |
| Configural invariance | 46.450 | 24 | .092 (.051, .131 | .982 | .031 |  |  |  |  |
| Metric invariance | 54.615 | 30 | .082 (.046, .116) | .982 | .048 | -.01 | .000 | .017 | .423 |
| Partial strong invariance | 65.194 | 35 | .082 (.050, .113) | .974 | .054 | .00 | -.003 | .006 | .051 |
|  |  |  |  |  |  |  |  |  |  |
| ***Body appreciation*** |  |  |  |  |  |  |  |  |  |
| Configural invariance | 114.341 | 58 | .090 (.066, .114) | .979 | .026 |  |  |  |  |
| Metric invariance | 134.389 | 67 | .088 (.067, .110) | .977 | .052 | -.002 | -.002 | .027 | .008* |
| Strong | 151.112 | 76 | .085 (.065, .105) | .976 | .055 | -.003 | -.001 | .002 | .073 |
| Strict | 162.818 | 86 | .083 (.063, .102) | .974 | .052 | -.002 | -.002 | -.003 | .185 |
|  |  |  |  |  |  |  |  |  |  |
| ***PYD*** |  |  |  |  |  |  |  |  |  |
| ***Competence*** |  |  |  |  |  |  |  |  |  |
| Configural invariance | 21.609 | 12 | .057 (.011, .094) | .985 | .036 |  |  |  |  |
| Metric invariance | 24.464 | 17 | .042 (.000, .076) | .989 | .042 | -.015 | .003 | .005 | .724 |
| Partial strong invariance | 27.757 | 20 | .039 (.000, .071) | .988 | .043 | -.003 | .000 | .001 | .359 |
|  |  |  |  |  |  |  |  |  |  |
| ***Confidence*** |  |  |  |  |  |  |  |  |  |
| Configural invariance | 6.192 | 4 | .045 (.000, .109) | .998 | .012 |  |  |  |  |
| Metric invariance | 12.681 | 7 | .055 (.000, .103) | .995 | .041 | .010 | -.003 | .029 | .094 |
| Strong invariance | 16.057 | 10 | .047 (.000, .089) | .994 | .043 | -.008 | -.001 | .002 | .349 |
|  |  |  |  |  |  |  |  |  |  |
| ***Connection*** |  |  |  |  |  |  |  |  |  |
| Configural invariance | 87.393 | 34 | .080 (.060, .101) | .972 | .079 |  |  |  |  |
| Metric invariance | 89.444 | 41 | .070 (.050, .090) | .974 | .080 | -.01 | .002 | .001 | .931 |
| Partial strong invariance | 99.614 | 46 | .069 (.050, .088) | .972 | .082 | -.001 | -.002 | .002 | .073 |
|  |  |  |  |  |  |  |  |  |  |
| ***Character*** |  |  |  |  |  |  |  |  |  |
| Configural invariance | 58.645 | 36 | .049 (.024, .071) | .974 | .041 |  |  |  |  |
| Metric invariance | 61.098 | 43 | .041 (.011, .063) | .978 | .045 | -.008 | .005 | .003 | .836 |
| Partial strong invariance | 70.901 | 48 | 0.43 (0.019, 0.063) | .973 | .048 | .002 | -.005 | .004 | .075 |
|  |  |  |  |  |  |  |  |  |  |
| ***Caring*** |  |  |  |  |  |  |  |  |  |
| Configural invariance | 24.374 | 14 | .063 (.013, .104) | .993 | .023 |  |  |  |  |
| Metric invariance | 29.506 | 19 | .053 (.000, .089) | .993 | .034 | -.001 | .00 | .011 | .456 |
| Strong invariance | 44.599 | 24 | .065 (.034, .094) | .987 | .042 | .011 | -.006 | .009 | .004* |
|  |  |  |  |  |  |  |  |  |  |
| ***General PYD*** |  |  |  |  |  |  |  |  |  |
| Configural invariance | 1573.973 | 828 | .060 (.055, .064) | .901 | .095 |  |  |  |  |
| Metric invariance | 1588.049 | 854 | .058 (.054, .063) | .903 | .096 | .001 | -.001 | .001 | .882 |
| *Chisquare test is significant, but the change in CFI is <.01, which according to Cheung & Rensvold (2002) is acceptable and supports invariance | | | | | | | | | |
