# Supplementary material for: Positive Youth Development and Mental Well-Being in Late Adolescence: The Role of Body Appreciation. Findings From a Prospective Study in Norway
Source: Front Psychol. 2021 Aug 23;12:696198. doi: 10.3389/fpsyg.2021.696198 (PMC8419256; doi:10.3389/fpsyg.2021.696198)
Supplement: Supplementary file 2 [file Table_2.docx]

| **Supplementary Table 2 Second stage moderated mediation models for Global PYD (T1) on mental well-being (T2) through body appreciation (T1), moderated by gender** | | | | |
| --- | --- | --- | --- | --- |
|  | Mental well-being at T2 | | | |
| Predictors | B | SE | z | *p* |
| Body appreciation | 0.08 | 0.08 | 0.96 | 0.335 |
| Global PYD | 0.10 | 0.04 | 2.430 | 0.015 |
| Gender | -0.51 | 0.39 | -1.292 | 0.196 |
| Body appreciation*Gender | 0.09 | 0.10 | 0.868 | 0.385 |
| Model summary | R2 = 0.308 |  |  |  |
|  | Conditional indirect effects at body appreciation | | | |
| Gender | B | Boot SE | Boot 95% CI | *p* |
| Male | -0.19 | 0.15 | -0.480, 0.096 | 0.202 |
| Female | -0.15 | 0.11 | -0.378, 0.052 | 0.162 |
| NOTE: B = unstandardised effect size. Bootstrap resamples = 5000. | | | | |
| Model adjusted for mental well-being at T1 and perceived family affluence | | |  |  |
