# Supplementary material for: Positive Youth Development and Mental Well-Being in Late Adolescence: The Role of Body Appreciation. Findings From a Prospective Study in Norway
Source: Front Psychol. 2021 Aug 23;12:696198. doi: 10.3389/fpsyg.2021.696198 (PMC8419256; doi:10.3389/fpsyg.2021.696198)
Supplement: Supplementary file 3 [file Table_3.docx]

| **Supplementary Table 3 Second stage moderated mediation models for Competence (T1) on mental well-being (T2) through body appreciation (T1), moderated by gender** | | | | |
| --- | --- | --- | --- | --- |
|  | Mental well-being at T2 | | | |
| Predictors | B | SE | z | *p* |
| Body appreciation | 0.08 | 0.09 | 0.881 | 0.379 |
| Competence | 0.06 | 0.03 | 1.915 | 0.056 |
| Gender | -0.685 | 0.45 | -1.523 | 0.128 |
| Body appreciation*Gender | 0.16 | 0.12 | 1.341 | 0.180 |
| Model summary | R2 = 0.257 |  |  |  |
|  | Conditional indirect effects at body appreciation | | | |
| Gender | B | Boot SE | Boot 95% CI | *p* |
| Male | -0.15 | 0.10 | -0.365, 0.039 | 0.142 |
| Female | -0.12 | 0.08 | -0.277, 0.024 | 0.132 |
| NOTE: B = unstandardised effect size. Bootstrap resamples = 5000. | | |  |  |
| Model adjusted for mental well-being at T1 and perceived family affluence | | | | |
