# Supplementary material for: Positive Youth Development and Mental Well-Being in Late Adolescence: The Role of Body Appreciation. Findings From a Prospective Study in Norway
Source: Front Psychol. 2021 Aug 23;12:696198. doi: 10.3389/fpsyg.2021.696198 (PMC8419256; doi:10.3389/fpsyg.2021.696198)
Supplement: Supplementary file 5 [file Table_5.docx]

| **Supplementary Table 5 Second stage moderated mediation models for Connection (T1) on mental well-being (T2) through body appreciation (T1), moderated by gender** | | | | |
| --- | --- | --- | --- | --- |
|  | Mental well-being at T2 | | | |
| Predictors | B | SE | z | *p* |
| Body appreciation | 0.07 | 0.09 | 0.763 | 0.446 |
| Connection | 0.08 | 0.03 | 2.477 | 0.013 |
| Gender | -0.53 | 0.46 | -1.170 | 0.242 |
| Body appreciation*Gender | 0.09 | 0.12 | 0.773 | 0.439 |
| Model summary | R2 = 0.264 |  |  |  |
|  | Conditional indirect effects at body appreciation | | | |
| Gender | B | Boot SE | Boot 95% CI | *p* |
| Male | -0.13 | 0.11 | -0.364, 0.081 | 0.265 |
| Female | -0.10 | 0.08 | -0.288, 0.048 | 0.219 |
| NOTE: B = unstandardised effect size. Bootstrap resamples = 5000. | | |  |  |
| Model adjusted for mental well-being at T1 and perceived family affluence | | | | |
