# Supplementary material for: Positive Youth Development and Mental Well-Being in Late Adolescence: The Role of Body Appreciation. Findings From a Prospective Study in Norway
Source: Front Psychol. 2021 Aug 23;12:696198. doi: 10.3389/fpsyg.2021.696198 (PMC8419256; doi:10.3389/fpsyg.2021.696198)
Supplement: Supplementary file 6 [file Table_6.docx]

| **Supplementary Table 6 Second stage moderated mediation models for Character (T1) on mental well-being (T2) through body appreciation (T1), moderated by gender** | | | | |
| --- | --- | --- | --- | --- |
|  | Mental well-being T2 | | | |
| Predictors | B | SE | z | *p* |
| Body appreciation | 0.10 | 0.10 | 1.086 | 0.278 |
| Character | 0.02 | 0.03 | 0.579 | 0.563 |
| Gender | -0.55 | 0.46 | -1.183 | 0.237 |
| Body appreciation*Gender | 0.11 | 0.12 | 0.901 | 0.368 |
| Model summary | R2 = 0.210 |  |  |  |
|  | Conditional indirect effects at body appreciation | | | |
| Gender | B | Boot SE | Boot 95% CI | *p* |
| Male | -0.07 | 0.07 | -0.217, 0.043 | 0.267 |
| Female | -0.06 | 0.05 | -0.168, 0.028 | 0.238 |
| NOTE: B = unstandardised effect size. Bootstrap resamples = 5000. | | |  |  |
| Model adjusted for mental well-being at T1 and perceived family affluence | | | | |
