# Supplementary material for: Positive Youth Development and Mental Well-Being in Late Adolescence: The Role of Body Appreciation. Findings From a Prospective Study in Norway
Source: Front Psychol. 2021 Aug 23;12:696198. doi: 10.3389/fpsyg.2021.696198 (PMC8419256; doi:10.3389/fpsyg.2021.696198)
Supplement: Supplementary file 7 [file Table_7.docx]

| **Supplementary Table 7 Second stage moderated mediation models for Caring (T1) on mental well-being (T2) through body appreciation (T1), moderated by gender** | | | | |
| --- | --- | --- | --- | --- |
|  | Mental well-being at T2 | | | |
| Predictors | B | SE | z | *p* |
| Body appreciation | 0.10 | 0.10 | 1.046 | 0.295 |
| Caring | 0.02 | 0.02 | 0.885 | 0.376 |
| Gender | -0.57 | 0.48 | -1.200 | 0.230 |
| Body appreciation*Gender | 0.11 | 0.12 | 0.883 | 0.377 |
| Model summary | R2 = 0.204 |  |  |  |
|  | Conditional indirect effects at body appreciation | | | |
| Gender | B | Boot SE | Boot 95% CI | *p* |
| Male | -0.03 | 0.03 | -0.112, 0.013 | 0.289 |
| Female | -0.03 | 0.02 | -0.087, 0.008 | 0.258 |
| NOTE: B = unstandardised effect size. Bootstrap resamples = 5000. | | | | |
| Model adjusted for mental well-being at T1 and perceived family affluence | | |  |  |
